# 8,14-Secogammacera-7,14(27)-diene-3,21-dione–8,14-secogammacera-7,14-diene-3,21-dione (1.5/0.5) from the bark of *Lansium domesticum* Corr.

**DOI:** 10.1107/S1600536809019710

**Published:** 2009-05-29

**Authors:** Roekmi-ati Tjokronegero, Tri Mayanti, Unang Supratman, Mat Ropi Mukhtar, Seik Weng Ng

**Affiliations:** a Department of Chemistry, Faculty of Mathematics and Natural Sciences, Padjadjaran University, Jatinangor 45363, Indonesia; b Department of Chemistry, University of Malaya, 50603 Kuala Lumpur, Malaysia

## Abstract

The components of the title cocrystal, 1.5C_30_H_40_O_2_·0.5C_30_H_40_O_2_, each have two tetra­hydro­deca­lin-type fused rings connected through an ethyl­ene fragment [*R*—CH_2_—CH_2_—*R*′ torsion angles: 158.1 (7) ° in the major component and 157.5 (6)° in the minor component]. The structure is a non-merohedral twin, with a minor twin component of 26%. The exocyclic double-bonded C atom of the major component of the cocrystal is disordered over two sites of equal occupancy.

## Related literature

For another compound from the seeds of this plant, see: Mayanti *et al.* (2009 ▶). For related compounds, see: Habaguchi *et al.* (1968 ▶); Kiang *et al.* (1967 ▶); Nishizawa *et al.* (1982 ▶, 1983 ▶, 1984 ▶); Tanaka *et al.* (2002 ▶). For the procedure to *TwinRotMat* the diffraction data, see: Spek (2003 ▶).
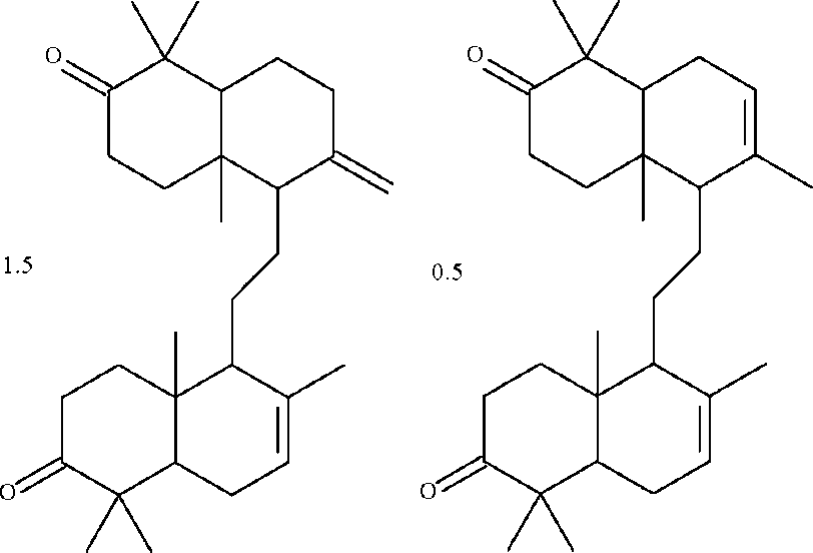


## Experimental

### 

#### Crystal data

1.5C_30_H_46_O_4_·0.5C_30_H_46_O_4_
*M* *_r_* = 877.34
Triclinic, 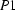

*a* = 8.4522 (2) Å
*b* = 11.7144 (2) Å
*c* = 14.1292 (3) Å
α = 107.484 (1)°
β = 90.249 (1)°
γ = 104.871 (1)°
*V* = 1284.60 (5) Å^3^
*Z* = 1
Mo *K*α radiation
μ = 0.07 mm^−1^
*T* = 118 K
0.42 × 0.08 × 0.06 mm

#### Data collection

Bruker SMART APEX diffractometer
Absorption correction: none
9450 measured reflections
4459 independent reflections
3954 reflections with *I* > 2σ(*I*)
*R*_int_ = 0.032

#### Refinement

*R*[*F* ^2^ > 2σ(*F* ^2^)] = 0.084
*wR*(*F* ^2^) = 0.249
*S* = 1.12
4459 reflections
603 parameters
66 restraints
H-atom parameters constrained
Δρ_max_ = 0.51 e Å^−3^
Δρ_min_ = −0.41 e Å^−3^

### 

Data collection: *APEX2* (Bruker, 2007 ▶); cell refinement: *SAINT* (Bruker, 2007 ▶); data reduction: *SAINT*; program(s) used to solve structure: *SHELXS97* (Sheldrick, 2008 ▶); program(s) used to refine structure: *SHELXL97* (Sheldrick, 2008 ▶); molecular graphics: *X-SEED* (Barbour, 2001 ▶); software used to prepare material for publication: *publCIF* (Westrip, 2009 ▶).

## Supplementary Material

Crystal structure: contains datablocks global, I. DOI: 10.1107/S1600536809019710/tk2453sup1.cif

Structure factors: contains datablocks I. DOI: 10.1107/S1600536809019710/tk2453Isup2.hkl

Additional supplementary materials:  crystallographic information; 3D view; checkCIF report

## Figures and Tables

**Fig. 1.**
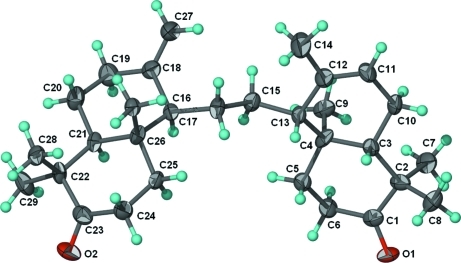
Thermal ellipsoid plot (Barbour, 2001) of the major component of co-crystal C30H40O2–C30H40O2 (1.5/0.5) (with the exocyclic double bond at the 14 position) at the 70% probability level. Hydrogen atoms are drawn as spheres of arbitrary radius.

**Fig. 2.**
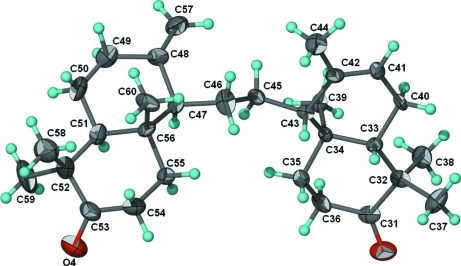
Thermal ellipsoid plot (Barbour, 2001) of the minor component of co-crystal C30H40O2–C30H40O2 (1.5/0.5) (with the endocyclic double bond at the 13 position) at the 70% probability level. Hydrogen atoms are drawn as spheres of arbitrary radius. The disorder is not shown.

## supplementary crystallographic information

### Experimental

*Lansium domesticum *Corr (Meliaceae) was collected in Cililin, Bandung, Indonesia, in 2006. The plant was identified by the staff at the Department of Biology, Padjadjaran University. The dried and milled bark of *L.domesticum* (3 kg) was extracted exhaustively by methanol at room temperature. The methanol extract (200 g) was partitioned between *n*-hexane and 10% aqueous methanol to give an *n*-hexane soluble fraction (70 g); 11 g of the *n*-hexane extract was subjected to column chromatography on silica gel 60 by using a step gradient of *n*-hexane and ethyl acetate (8:2) followed by separation with preparative thin layer chromatography on silica gel. The co-crystal was eluted with an ethyl acetate: *n-*hexane (9:1) mixture; onoceradienedione (33 mg) was obtained as a powder along with several crystals when the solvent was allowed to evaporate.

The formulation of the co-crystal, *i.e.*, a 75% component having an excocyclic double bond and a 25% component having an endocyclic double bond, was established by NMR spectroscopic analysis.

### Refinement

Carbon-bound H-atoms were placed in calculated positions (C–H 0.95–1.00 Å) and were included in the refinement in the riding model approximation, with *U*(H) set to 1.2–1.5*U*(C).

The structure is a non-merohdral twin. The twin law, as given by *PLATON* (Spek, 2003), is (1.010 0.030 0.013, -0.670 - 1.010 - 0.004, 0.000 0.000 - 1.000), which lowered the *R*_1_ index from 10.4% to 8.40%. A total of X Friedel pairs were merged.

The exocyclic double-bonded carbon atom of major component of the co-crystal is disordered over two sites (the ring carbon is the carbon atom at the 14 position according to IUPAC nomenclature); the occupancy was arbitrarily set as 50:50. The disorder required the ring carbon at 13 position to be disordered. The exocyclic carbon-carbon double-bond distance was restrained to 1.35±0.01 Å (in the umprimed fragment) and the exocyclic carbon-carbon single bond was restrained to 1.54±0.01 Å (in the primed fragment). The endocylic single- and double-bond distances were similarly restrained. The anisotropic displacement parameters of the primed atoms were set to those of the umprimed ones, and these were restrained to be nearly isotropic.

The disorder also affected the two methyl groups at carbon atom adjacent to the carbonyl group at the 21 position. For the four carbon atoms connected to this atom, the 1,2-related distances were restrained to 1.54±0.01 Å and the 1,3-related ones to 2.51±0.01 Å. The anisotropic displacement parameters of all five atoms were restrained to be nearly isotropic. Finally, the distance between the H46*b* and H57d atoms was restrained to 2.00±0.01 Å; this additional restraint was applied to the C57 methyl group.

The assignment of the atoms was guided by NMR spectral measurements. The ratio of the two chemically similar molecules were furnished by proton NMR spectral integration.

### Crystal data

**Table:**

1.5C_30_H_46_O_4_·0.5C_30_H_46_O_4_	*Z* = 1
*M**_r_* = 877.34	*F*(000) = 484
Triclinic, *P*1	*D*_x_ = 1.134 Mg m^−^^3^
Hall symbol: P 1	Mo *K*α radiation, λ = 0.71073 Å
*a* = 8.4522 (2) Å	Cell parameters from 4429 reflections
*b* = 11.7144 (2) Å	θ = 2.5–28.2°
*c* = 14.1292 (3) Å	µ = 0.07 mm^−^^1^
α = 107.484 (1)°	*T* = 118 K
β = 90.249 (1)°	Irregular fragment, colorless
γ = 104.871 (1)°	0.42 × 0.08 × 0.06 mm
*V* = 1284.60 (5) Å^3^	

### Data collection

**Table:**

Bruker SMART APEX diffractometer	3954 reflections with *I* > 2σ(*I*)
Radiation source: fine-focus sealed tube	*R*_int_ = 0.032
graphite	θ_max_ = 25.0°, θ_min_ = 1.5°
ω scans	*h* = −10→10
9450 measured reflections	*k* = −13→13
4459 independent reflections	*l* = −16→16

### Refinement

**Table:**

Refinement on *F*^2^	Hydrogen site location: inferred from neighbouring sites
Least-squares matrix: full	H-atom parameters constrained
*R*[*F*^2^ > 2σ(*F*^2^)] = 0.084	*w* = 1/[σ^2^(*F*_o_^2^) + (0.1369*P*)^2^ + 1.7886*P*] where *P* = (*F*_o_^2^ + 2*F*_c_^2^)/3
*wR*(*F*^2^) = 0.249	(Δ/σ)_max_ = 0.001
*S* = 1.12	Δρ_max_ = 0.51 e Å^−^^3^
4459 reflections	Δρ_min_ = −0.41 e Å^−^^3^
603 parameters	Extinction correction: *SHELXL97* (Sheldrick, 2008), Fc^*^=kFc[1+0.001xFc^2^λ^3^/sin(2θ)]^-1/4^
66 restraints	Extinction coefficient: 0.034 (9)
Primary atom site location: structure-invariant direct methods	Absolute structure: Friedel pairs were merged
Secondary atom site location: difference Fourier map	

### Fractional atomic coordinates and isotropic or equivalent isotropic displacement parameters (Å^2^)

**Table:**

	*x*	*y*	*z*	*U*_iso_*/*U*_eq_	Occ. (<1)
O1	0.4978 (7)	0.4989 (6)	0.5001 (4)	0.0358 (14)	
O2	0.8406 (8)	0.2884 (7)	−0.2153 (5)	0.0456 (16)	
O3	0.6627 (8)	−0.0020 (6)	0.8831 (4)	0.0416 (15)	
O4	0.2865 (10)	0.1987 (9)	0.2699 (6)	0.065 (2)	
C1	0.4206 (10)	0.5309 (7)	0.4436 (5)	0.0263 (16)	
C2	0.2372 (10)	0.5138 (7)	0.4471 (5)	0.0270 (16)	
C3	0.1500 (9)	0.4964 (7)	0.3425 (5)	0.0252 (16)	
H3	0.1275	0.4055	0.3065	0.030*	
C4	0.2476 (10)	0.5601 (7)	0.2708 (5)	0.0247 (16)	
C5	0.4184 (9)	0.5382 (7)	0.2690 (5)	0.0269 (16)	
H5A	0.4077	0.4479	0.2432	0.032*	
H5B	0.4826	0.5780	0.2234	0.032*	
C6	0.5098 (9)	0.5913 (8)	0.3728 (6)	0.0292 (17)	
H6A	0.6211	0.5785	0.3687	0.035*	
H6B	0.5219	0.6819	0.3981	0.035*	
C7	0.2225 (11)	0.6324 (8)	0.5296 (6)	0.0343 (19)	
H7A	0.2799	0.6389	0.5922	0.051*	
H7B	0.1063	0.6270	0.5390	0.051*	
H7C	0.2720	0.7060	0.5097	0.051*	
C8	0.1579 (11)	0.4018 (8)	0.4801 (6)	0.0360 (19)	
H8A	0.1922	0.4196	0.5506	0.054*	
H8B	0.1924	0.3296	0.4401	0.054*	
H8C	0.0380	0.3839	0.4710	0.054*	
C9	0.2587 (11)	0.6997 (7)	0.3003 (6)	0.0306 (17)	
H9A	0.2903	0.7380	0.3719	0.046*	
H9B	0.1516	0.7106	0.2845	0.046*	
H9C	0.3413	0.7395	0.2633	0.046*	
C10	−0.0194 (10)	0.5195 (8)	0.3499 (5)	0.0312 (18)	
H10A	−0.0876	0.4650	0.3846	0.037*	
H10B	−0.0082	0.6068	0.3903	0.037*	
C11	−0.1049 (10)	0.4950 (9)	0.2495 (6)	0.036 (2)	
H11	−0.2187	0.4902	0.2452	0.044*	
C12	−0.0271 (10)	0.4793 (8)	0.1648 (6)	0.0304 (18)	
C13	0.1536 (9)	0.4923 (7)	0.1643 (5)	0.0223 (15)	
H13	0.1665	0.4059	0.1437	0.027*	
C14	−0.1296 (11)	0.4461 (11)	0.0675 (7)	0.048 (2)	
H14A	−0.2463	0.4287	0.0791	0.072*	
H14B	−0.1086	0.3723	0.0200	0.072*	
H14C	−0.1002	0.5157	0.0403	0.072*	
C15	0.2347 (10)	0.5529 (7)	0.0862 (5)	0.0256 (16)	
H15A	0.1747	0.6122	0.0780	0.031*	
H15B	0.3492	0.6008	0.1121	0.031*	
C16	0.2363 (9)	0.4589 (7)	−0.0161 (5)	0.0252 (16)	
H16A	0.2553	0.3830	−0.0065	0.030*	
H16B	0.1272	0.4351	−0.0533	0.030*	
C17	0.3681 (8)	0.5090 (6)	−0.0784 (5)	0.0199 (14)	
H17	0.4691	0.5536	−0.0315	0.024*	
C18	0.3311 (10)	0.6044 (7)	−0.1215 (5)	0.0263 (16)	
C19	0.4661 (11)	0.6623 (8)	−0.1720 (7)	0.0348 (18)	
H19A	0.5598	0.7138	−0.1222	0.042*	
H19B	0.4287	0.7176	−0.2026	0.042*	
C20	0.5233 (11)	0.5650 (7)	−0.2525 (6)	0.0313 (18)	
H20A	0.6198	0.6065	−0.2810	0.038*	
H20B	0.4344	0.5202	−0.3068	0.038*	
C21	0.5690 (9)	0.4728 (6)	−0.2079 (5)	0.0206 (14)	
H21	0.6485	0.5257	−0.1490	0.025*	
C22	0.6670 (9)	0.3905 (7)	−0.2771 (5)	0.0243 (16)	
C23	0.7036 (10)	0.3031 (7)	−0.2234 (6)	0.0290 (17)	
C24	0.5618 (10)	0.2319 (7)	−0.1828 (6)	0.0296 (17)	
H24A	0.6020	0.1844	−0.1453	0.035*	
H24B	0.4833	0.1719	−0.2387	0.035*	
C25	0.4741 (9)	0.3192 (7)	−0.1143 (5)	0.0231 (15)	
H25A	0.5491	0.3712	−0.0542	0.028*	
H25B	0.3771	0.2687	−0.0925	0.028*	
C26	0.4176 (9)	0.4046 (6)	−0.1630 (5)	0.0209 (15)	
C27	0.1888 (11)	0.6331 (9)	−0.1156 (7)	0.040 (2)	
H27A	0.1713	0.6928	−0.1446	0.048*	
H27B	0.1044	0.5940	−0.0825	0.048*	
C28	0.5751 (10)	0.3063 (7)	−0.3786 (5)	0.0282 (17)	
H28A	0.6497	0.2649	−0.4196	0.042*	
H28B	0.5362	0.3569	−0.4126	0.042*	
H28C	0.4810	0.2437	−0.3680	0.042*	
C29	0.8282 (10)	0.4723 (8)	−0.2964 (7)	0.038 (2)	
H29A	0.8987	0.4200	−0.3285	0.056*	
H29B	0.8843	0.5312	−0.2330	0.056*	
H29C	0.8048	0.5182	−0.3400	0.056*	
C30	0.2681 (9)	0.3305 (6)	−0.2379 (5)	0.0207 (14)	
H30A	0.2929	0.2578	−0.2848	0.031*	
H30B	0.2418	0.3832	−0.2746	0.031*	
H30C	0.1739	0.3029	−0.2023	0.031*	
C31	0.7311 (10)	−0.0360 (7)	0.8087 (6)	0.0261 (16)	
C32	0.9146 (9)	−0.0262 (7)	0.8147 (6)	0.0229 (15)	
C33	0.9937 (8)	−0.0113 (6)	0.7161 (5)	0.0186 (14)	
H33	1.0210	0.0799	0.7256	0.022*	
C34	0.8886 (9)	−0.0729 (6)	0.6156 (5)	0.0193 (14)	
C35	0.7205 (9)	−0.0454 (7)	0.6294 (5)	0.0246 (16)	
H35A	0.7357	0.0456	0.6495	0.030*	
H35B	0.6513	−0.0834	0.5653	0.030*	
C36	0.6330 (9)	−0.0967 (8)	0.7086 (6)	0.0289 (17)	
H36A	0.5230	−0.0813	0.7136	0.035*	
H36B	0.6177	−0.1877	0.6887	0.035*	
C37	1.0002 (12)	0.0875 (8)	0.9011 (6)	0.037 (2)	
H37A	0.9618	0.0760	0.9638	0.055*	
H37B	0.9749	0.1612	0.8928	0.055*	
H37C	1.1193	0.0987	0.9024	0.055*	
C38	0.9304 (11)	−0.1429 (7)	0.8389 (6)	0.0303 (17)	
H38A	0.8772	−0.1471	0.8997	0.045*	
H38B	1.0469	−0.1385	0.8489	0.045*	
H38C	0.8771	−0.2172	0.7835	0.045*	
C39	0.8652 (9)	−0.2147 (6)	0.5766 (5)	0.0226 (15)	
H39A	0.8354	−0.2502	0.6307	0.034*	
H39B	0.9678	−0.2316	0.5522	0.034*	
H39C	0.7775	−0.2523	0.5221	0.034*	
C40	1.1577 (8)	−0.0420 (7)	0.7075 (5)	0.0225 (15)	
H40A	1.2322	0.0106	0.7676	0.027*	
H40B	1.1412	−0.1300	0.7042	0.027*	
C41	1.2368 (8)	−0.0216 (7)	0.6165 (5)	0.0229 (15)	
H41	1.3492	−0.0207	0.6119	0.027*	
C42	1.1577 (8)	−0.0047 (6)	0.5418 (5)	0.0203 (14)	
C43	0.9770 (8)	−0.0119 (6)	0.5391 (5)	0.0175 (14)	
H43	0.9690	0.0758	0.5603	0.021*	
C44	1.2528 (10)	0.0256 (8)	0.4583 (6)	0.0319 (17)	
H44A	1.3693	0.0333	0.4726	0.048*	
H44B	1.2404	0.1042	0.4526	0.048*	
H44C	1.2104	−0.0410	0.3956	0.048*	
C45	0.8865 (8)	−0.0753 (6)	0.4324 (5)	0.0203 (14)	
H45A	0.9389	−0.1400	0.3953	0.024*	
H45B	0.7712	−0.1176	0.4384	0.024*	
C46	0.8863 (10)	0.0133 (8)	0.3714 (6)	0.0316 (18)	
H46A	0.8766	0.0937	0.4167	0.038*	
H46B	0.9928	0.0293	0.3421	0.038*	
C47	0.7467 (9)	−0.0368 (7)	0.2873 (5)	0.0215 (15)	
H47	0.6471	−0.0779	0.3149	0.026*	0.50
H47'	0.6473	−0.0766	0.3158	0.026*	0.50
C48	0.786 (4)	−0.142 (2)	0.198 (3)	0.032 (3)	0.50
C49	0.677 (4)	−0.165 (2)	0.1035 (18)	0.032 (4)	0.50
H49A	0.7501	−0.1718	0.0489	0.038*	0.50
H49B	0.6007	−0.2487	0.0908	0.038*	0.50
C48'	0.763 (3)	−0.129 (3)	0.198 (3)	0.032 (3)	0.50
C49'	0.646 (3)	−0.189 (2)	0.1219 (18)	0.032 (4)	0.50
H49'	0.6088	−0.2772	0.0933	0.038*	0.50
C50	0.5790 (12)	−0.0908 (9)	0.0873 (6)	0.042 (2)	
H50A	0.4733	−0.1467	0.0514	0.050*	0.50
H50B	0.6343	−0.0454	0.0425	0.050*	0.50
H50C	0.6627	−0.0479	0.0516	0.050*	0.50
H50D	0.4783	−0.1342	0.0413	0.050*	0.50
C51	0.5411 (9)	0.0035 (6)	0.1783 (5)	0.0297 (17)	
H51	0.4662	−0.0489	0.2128	0.036*	
C52	0.4376 (9)	0.0838 (7)	0.1550 (5)	0.040 (2)	
C53	0.4158 (10)	0.1782 (7)	0.2527 (5)	0.0352 (19)	
C54	0.5677 (11)	0.2462 (9)	0.3207 (6)	0.0370 (19)	
H54A	0.5393	0.3011	0.3826	0.044*	
H54B	0.6443	0.2994	0.2885	0.044*	
C55	0.6537 (9)	0.1592 (7)	0.3472 (5)	0.0258 (16)	
H55A	0.5802	0.1103	0.3838	0.031*	
H55B	0.7541	0.2092	0.3919	0.031*	
C56	0.7009 (9)	0.0698 (6)	0.2548 (5)	0.0212 (15)	
C57	0.844 (2)	−0.2388 (15)	0.1999 (12)	0.035 (3)	0.50
H57A	0.8162	−0.3136	0.1454	0.042*	0.50
H57B	0.9128	−0.2319	0.2558	0.042*	0.50
C57'	0.935 (2)	−0.1516 (16)	0.1936 (12)	0.035 (3)	0.50
H57C	0.9524	−0.1877	0.2456	0.053*	0.50
H57D	1.0186	−0.0726	0.2044	0.053*	0.50
H57E	0.9425	−0.2091	0.1282	0.053*	0.50
C58	0.5217 (12)	0.1574 (8)	0.0869 (6)	0.053 (3)	
H58A	0.6261	0.2150	0.1208	0.080*	
H58B	0.4500	0.2045	0.0715	0.080*	
H58C	0.5424	0.0997	0.0249	0.080*	
C59	0.2678 (11)	0.0000 (10)	0.1045 (8)	0.081 (4)	
H59A	0.2021	0.0513	0.0896	0.121*	
H59B	0.2115	−0.0435	0.1494	0.121*	
H59C	0.2820	−0.0609	0.0425	0.121*	
C60	0.8467 (11)	0.1366 (8)	0.2113 (7)	0.0333 (18)	
H60A	0.8204	0.2054	0.1947	0.050*	
H60B	0.8709	0.0782	0.1509	0.050*	
H60C	0.9427	0.1692	0.2603	0.050*	

### Atomic displacement parameters (Å^2^)

**Table:**

	*U*^11^	*U*^22^	*U*^33^	*U*^12^	*U*^13^	*U*^23^
O1	0.036 (3)	0.052 (4)	0.030 (3)	0.023 (3)	−0.003 (2)	0.018 (3)
O2	0.025 (3)	0.066 (4)	0.061 (4)	0.024 (3)	0.012 (3)	0.032 (3)
O3	0.048 (4)	0.057 (4)	0.037 (3)	0.028 (3)	0.025 (3)	0.027 (3)
O4	0.055 (5)	0.100 (6)	0.053 (4)	0.052 (4)	0.009 (4)	0.018 (4)
C1	0.034 (4)	0.022 (4)	0.022 (4)	0.014 (3)	−0.001 (3)	0.001 (3)
C2	0.028 (4)	0.032 (4)	0.022 (4)	0.015 (3)	−0.002 (3)	0.003 (3)
C3	0.024 (4)	0.034 (4)	0.018 (3)	0.013 (3)	0.003 (3)	0.006 (3)
C4	0.037 (4)	0.024 (4)	0.012 (3)	0.008 (3)	−0.005 (3)	0.005 (3)
C5	0.025 (4)	0.034 (4)	0.022 (4)	0.009 (3)	0.002 (3)	0.008 (3)
C6	0.017 (4)	0.041 (4)	0.029 (4)	0.003 (3)	0.001 (3)	0.014 (3)
C7	0.047 (5)	0.038 (5)	0.019 (4)	0.021 (4)	0.002 (3)	0.002 (3)
C8	0.041 (5)	0.047 (5)	0.024 (4)	0.014 (4)	0.000 (3)	0.017 (4)
C9	0.039 (5)	0.029 (4)	0.025 (4)	0.015 (3)	−0.002 (3)	0.006 (3)
C10	0.032 (4)	0.049 (5)	0.018 (4)	0.016 (4)	0.007 (3)	0.013 (3)
C11	0.018 (4)	0.067 (6)	0.029 (4)	0.015 (4)	0.005 (3)	0.018 (4)
C12	0.025 (4)	0.044 (5)	0.023 (4)	0.010 (4)	0.001 (3)	0.012 (3)
C13	0.021 (4)	0.031 (4)	0.014 (3)	0.007 (3)	0.002 (3)	0.006 (3)
C14	0.029 (5)	0.086 (8)	0.028 (4)	0.011 (5)	−0.001 (4)	0.019 (5)
C15	0.030 (4)	0.026 (4)	0.021 (4)	0.008 (3)	0.003 (3)	0.008 (3)
C16	0.028 (4)	0.028 (4)	0.016 (3)	0.002 (3)	0.003 (3)	0.006 (3)
C17	0.017 (3)	0.025 (4)	0.015 (3)	0.003 (3)	−0.001 (3)	0.004 (3)
C18	0.035 (4)	0.024 (4)	0.021 (4)	0.012 (3)	0.001 (3)	0.004 (3)
C19	0.037 (5)	0.030 (4)	0.037 (4)	0.008 (3)	0.010 (4)	0.011 (3)
C20	0.039 (5)	0.024 (4)	0.031 (4)	0.004 (3)	0.010 (3)	0.013 (3)
C21	0.021 (4)	0.021 (3)	0.015 (3)	−0.001 (3)	0.001 (3)	0.004 (3)
C22	0.022 (4)	0.024 (4)	0.025 (4)	−0.001 (3)	0.009 (3)	0.010 (3)
C23	0.027 (4)	0.032 (4)	0.026 (4)	0.011 (3)	0.002 (3)	0.002 (3)
C24	0.028 (4)	0.031 (4)	0.036 (4)	0.009 (3)	0.007 (3)	0.017 (3)
C25	0.024 (4)	0.029 (4)	0.018 (3)	0.005 (3)	0.003 (3)	0.011 (3)
C26	0.027 (4)	0.018 (3)	0.013 (3)	0.002 (3)	−0.001 (3)	0.003 (3)
C27	0.035 (5)	0.059 (6)	0.041 (5)	0.021 (4)	0.011 (4)	0.030 (4)
C28	0.039 (5)	0.029 (4)	0.019 (3)	0.015 (3)	0.006 (3)	0.006 (3)
C29	0.025 (4)	0.039 (5)	0.050 (5)	0.007 (4)	0.019 (4)	0.016 (4)
C30	0.020 (4)	0.019 (3)	0.021 (3)	0.003 (3)	0.003 (3)	0.006 (3)
C31	0.036 (4)	0.026 (4)	0.028 (4)	0.015 (3)	0.014 (3)	0.019 (3)
C32	0.022 (4)	0.020 (3)	0.030 (4)	0.006 (3)	0.006 (3)	0.011 (3)
C33	0.017 (3)	0.020 (3)	0.018 (3)	0.004 (3)	0.003 (3)	0.005 (3)
C34	0.024 (4)	0.023 (4)	0.017 (3)	0.014 (3)	0.001 (3)	0.008 (3)
C35	0.026 (4)	0.032 (4)	0.021 (4)	0.014 (3)	0.003 (3)	0.011 (3)
C36	0.018 (4)	0.043 (4)	0.035 (4)	0.013 (3)	0.007 (3)	0.022 (4)
C37	0.051 (5)	0.033 (4)	0.021 (4)	0.003 (4)	0.006 (4)	0.009 (3)
C38	0.040 (5)	0.026 (4)	0.031 (4)	0.012 (3)	0.001 (3)	0.016 (3)
C39	0.023 (4)	0.023 (4)	0.020 (3)	0.004 (3)	−0.002 (3)	0.006 (3)
C40	0.013 (3)	0.035 (4)	0.023 (4)	0.010 (3)	−0.001 (3)	0.009 (3)
C41	0.012 (3)	0.031 (4)	0.022 (4)	0.002 (3)	−0.001 (3)	0.004 (3)
C42	0.016 (3)	0.019 (3)	0.022 (3)	0.003 (3)	0.005 (3)	0.003 (3)
C43	0.017 (3)	0.015 (3)	0.021 (3)	0.005 (3)	0.005 (3)	0.007 (3)
C44	0.027 (4)	0.037 (4)	0.031 (4)	0.004 (3)	0.006 (3)	0.014 (3)
C45	0.017 (3)	0.024 (4)	0.023 (4)	0.007 (3)	0.004 (3)	0.011 (3)
C46	0.028 (4)	0.038 (4)	0.034 (4)	0.000 (3)	0.003 (3)	0.026 (4)
C47	0.022 (4)	0.030 (4)	0.016 (3)	0.006 (3)	0.006 (3)	0.012 (3)
C48	0.055 (7)	0.035 (6)	0.026 (4)	0.034 (4)	0.011 (5)	0.018 (4)
C49	0.038 (8)	0.022 (8)	0.031 (8)	0.002 (6)	0.002 (6)	0.005 (5)
C48'	0.055 (7)	0.035 (6)	0.026 (4)	0.034 (4)	0.011 (5)	0.018 (4)
C49'	0.038 (8)	0.022 (8)	0.031 (8)	0.002 (6)	0.002 (6)	0.005 (5)
C50	0.051 (5)	0.052 (5)	0.020 (4)	0.018 (4)	−0.006 (4)	0.004 (4)
C51	0.031 (4)	0.034 (4)	0.021 (3)	0.008 (3)	−0.001 (3)	0.007 (3)
C52	0.043 (5)	0.059 (5)	0.026 (4)	0.025 (4)	−0.001 (3)	0.013 (4)
C53	0.036 (4)	0.051 (5)	0.034 (4)	0.027 (4)	0.009 (3)	0.023 (4)
C54	0.038 (5)	0.046 (5)	0.031 (4)	0.023 (4)	0.009 (4)	0.007 (4)
C55	0.021 (4)	0.034 (4)	0.022 (4)	0.009 (3)	0.004 (3)	0.006 (3)
C56	0.025 (4)	0.025 (4)	0.016 (3)	0.009 (3)	0.007 (3)	0.008 (3)
C57	0.044 (6)	0.035 (5)	0.027 (5)	0.016 (5)	0.007 (5)	0.005 (4)
C57'	0.044 (6)	0.035 (5)	0.027 (5)	0.016 (5)	0.007 (5)	0.005 (4)
C58	0.067 (5)	0.069 (5)	0.043 (4)	0.043 (4)	0.009 (4)	0.025 (4)
C59	0.063 (8)	0.092 (9)	0.075 (8)	0.039 (7)	−0.043 (7)	−0.005 (7)
C60	0.038 (5)	0.040 (5)	0.041 (4)	0.027 (4)	0.015 (4)	0.028 (4)

### Geometric parameters (Å, °)

**Table:**

O1—C1	1.227 (9)	C32—C33	1.585 (10)
O2—C23	1.224 (10)	C33—C40	1.516 (9)
O3—C31	1.212 (9)	C33—C34	1.540 (9)
O4—C53	1.189 (10)	C33—H33	1.0000
C1—C6	1.493 (11)	C34—C35	1.537 (10)
C1—C2	1.514 (11)	C34—C39	1.543 (10)
C2—C8	1.520 (12)	C34—C43	1.560 (9)
C2—C7	1.556 (10)	C35—C36	1.532 (10)
C2—C3	1.581 (10)	C35—H35A	0.9900
C3—C10	1.522 (11)	C35—H35B	0.9900
C3—C4	1.552 (10)	C36—H36A	0.9900
C3—H3	1.0000	C36—H36B	0.9900
C4—C5	1.529 (11)	C37—H37A	0.9800
C4—C9	1.539 (11)	C37—H37B	0.9800
C4—C13	1.573 (9)	C37—H37C	0.9800
C5—C6	1.526 (10)	C38—H38A	0.9800
C5—H5A	0.9900	C38—H38B	0.9800
C5—H5B	0.9900	C38—H38C	0.9800
C6—H6A	0.9900	C39—H39A	0.9800
C6—H6B	0.9900	C39—H39B	0.9800
C7—H7A	0.9800	C39—H39C	0.9800
C7—H7B	0.9800	C40—C41	1.506 (10)
C7—H7C	0.9800	C40—H40A	0.9900
C8—H8A	0.9800	C40—H40B	0.9900
C8—H8B	0.9800	C41—C42	1.337 (10)
C8—H8C	0.9800	C41—H41	0.9500
C9—H9A	0.9800	C42—C43	1.508 (9)
C9—H9B	0.9800	C42—C44	1.508 (10)
C9—H9C	0.9800	C43—C45	1.560 (10)
C10—C11	1.502 (11)	C43—H43	1.0000
C10—H10A	0.9900	C44—H44A	0.9800
C10—H10B	0.9900	C44—H44B	0.9800
C11—C12	1.355 (11)	C44—H44C	0.9800
C11—H11	0.9500	C45—C46	1.537 (10)
C12—C13	1.496 (11)	C45—H45A	0.9900
C12—C14	1.512 (11)	C45—H45B	0.9900
C13—C15	1.555 (10)	C46—C47	1.537 (10)
C13—H13	1.0000	C46—H46A	0.9900
C14—H14A	0.9800	C46—H46B	0.9900
C14—H14B	0.9800	C47—C48'	1.43 (3)
C14—H14C	0.9800	C47—C48	1.59 (3)
C15—C16	1.535 (10)	C47—C56	1.585 (9)
C15—H15A	0.9900	C47—H47	1.0000
C15—H15B	0.9900	C47—H47'	1.0000
C16—C17	1.531 (10)	C48—C57	1.352 (10)
C16—H16A	0.9900	C48—C49	1.534 (10)
C16—H16B	0.9900	C49—C50	1.40 (3)
C17—C18	1.518 (10)	C49—H49A	0.9900
C17—C26	1.577 (9)	C49—H49B	0.9900
C17—H17	1.0000	C48'—C49'	1.342 (10)
C18—C27	1.326 (12)	C48'—C57'	1.540 (10)
C18—C19	1.474 (11)	C49'—C50	1.60 (3)
C19—C20	1.528 (11)	C49'—H49'	0.9500
C19—H19A	0.9900	C50—C51	1.518 (11)
C19—H19B	0.9900	C50—H50A	0.9900
C20—C21	1.528 (10)	C50—H50B	0.9900
C20—H20A	0.9900	C50—H50C	0.9900
C20—H20B	0.9900	C50—H50D	0.9900
C21—C22	1.556 (10)	C51—C52	1.538 (7)
C21—C26	1.570 (9)	C51—C56	1.594 (10)
C21—H21	1.0000	C51—H51	1.0000
C22—C29	1.529 (10)	C52—C53	1.534 (7)
C22—C23	1.533 (11)	C52—C58	1.538 (8)
C22—C28	1.542 (10)	C52—C59	1.542 (8)
C23—C24	1.496 (11)	C53—C54	1.487 (12)
C24—C25	1.527 (11)	C54—C55	1.521 (11)
C24—H24A	0.9900	C54—H54A	0.9900
C24—H24B	0.9900	C54—H54B	0.9900
C25—C26	1.537 (10)	C55—C56	1.535 (9)
C25—H25A	0.9900	C55—H55A	0.9900
C25—H25B	0.9900	C55—H55B	0.9900
C26—C30	1.531 (9)	C56—C60	1.519 (11)
C27—H27A	0.9500	C57—H57A	0.9500
C27—H27B	0.9500	C57—H57B	0.9500
C28—H28A	0.9800	C57'—H57C	0.9800
C28—H28B	0.9800	C57'—H57D	0.9800
C28—H28C	0.9800	C57'—H57E	0.9800
C29—H29A	0.9800	C58—H58A	0.9800
C29—H29B	0.9800	C58—H58B	0.9800
C29—H29C	0.9800	C58—H58C	0.9800
C30—H30A	0.9800	C59—H59A	0.9800
C30—H30B	0.9800	C59—H59B	0.9800
C30—H30C	0.9800	C59—H59C	0.9800
C31—C36	1.508 (11)	C60—H60A	0.9800
C31—C32	1.525 (11)	C60—H60B	0.9800
C32—C37	1.519 (11)	C60—H60C	0.9800
C32—C38	1.542 (10)		
			
O1—C1—C6	119.5 (7)	C35—C34—C33	108.1 (6)
O1—C1—C2	120.9 (7)	C35—C34—C39	109.8 (6)
C6—C1—C2	119.6 (6)	C33—C34—C39	112.5 (5)
C1—C2—C8	110.8 (7)	C35—C34—C43	109.2 (5)
C1—C2—C7	104.1 (6)	C33—C34—C43	107.7 (6)
C8—C2—C7	108.0 (7)	C39—C34—C43	109.5 (5)
C1—C2—C3	112.0 (6)	C36—C35—C34	111.2 (6)
C8—C2—C3	108.7 (6)	C36—C35—H35A	109.4
C7—C2—C3	113.1 (6)	C34—C35—H35A	109.4
C10—C3—C4	110.2 (6)	C36—C35—H35B	109.4
C10—C3—C2	113.0 (6)	C34—C35—H35B	109.4
C4—C3—C2	119.1 (6)	H35A—C35—H35B	108.0
C10—C3—H3	104.3	C31—C36—C35	110.8 (6)
C4—C3—H3	104.3	C31—C36—H36A	109.5
C2—C3—H3	104.3	C35—C36—H36A	109.5
C5—C4—C9	111.2 (6)	C31—C36—H36B	109.5
C5—C4—C3	108.1 (6)	C35—C36—H36B	109.5
C9—C4—C3	112.5 (6)	H36A—C36—H36B	108.1
C5—C4—C13	108.8 (6)	C32—C37—H37A	109.5
C9—C4—C13	109.5 (6)	C32—C37—H37B	109.5
C3—C4—C13	106.5 (6)	H37A—C37—H37B	109.5
C6—C5—C4	111.3 (6)	C32—C37—H37C	109.5
C6—C5—H5A	109.4	H37A—C37—H37C	109.5
C4—C5—H5A	109.4	H37B—C37—H37C	109.5
C6—C5—H5B	109.4	C32—C38—H38A	109.5
C4—C5—H5B	109.4	C32—C38—H38B	109.5
H5A—C5—H5B	108.0	H38A—C38—H38B	109.5
C1—C6—C5	111.4 (6)	C32—C38—H38C	109.5
C1—C6—H6A	109.3	H38A—C38—H38C	109.5
C5—C6—H6A	109.3	H38B—C38—H38C	109.5
C1—C6—H6B	109.3	C34—C39—H39A	109.5
C5—C6—H6B	109.3	C34—C39—H39B	109.5
H6A—C6—H6B	108.0	H39A—C39—H39B	109.5
C2—C7—H7A	109.5	C34—C39—H39C	109.5
C2—C7—H7B	109.5	H39A—C39—H39C	109.5
H7A—C7—H7B	109.5	H39B—C39—H39C	109.5
C2—C7—H7C	109.5	C41—C40—C33	111.9 (6)
H7A—C7—H7C	109.5	C41—C40—H40A	109.2
H7B—C7—H7C	109.5	C33—C40—H40A	109.2
C2—C8—H8A	109.5	C41—C40—H40B	109.2
C2—C8—H8B	109.5	C33—C40—H40B	109.2
H8A—C8—H8B	109.5	H40A—C40—H40B	107.9
C2—C8—H8C	109.5	C42—C41—C40	123.7 (6)
H8A—C8—H8C	109.5	C42—C41—H41	118.2
H8B—C8—H8C	109.5	C40—C41—H41	118.2
C4—C9—H9A	109.5	C41—C42—C43	122.3 (6)
C4—C9—H9B	109.5	C41—C42—C44	119.1 (6)
H9A—C9—H9B	109.5	C43—C42—C44	118.6 (6)
C4—C9—H9C	109.5	C42—C43—C45	113.1 (6)
H9A—C9—H9C	109.5	C42—C43—C34	112.2 (6)
H9B—C9—H9C	109.5	C45—C43—C34	111.5 (5)
C11—C10—C3	112.3 (6)	C42—C43—H43	106.5
C11—C10—H10A	109.1	C45—C43—H43	106.5
C3—C10—H10A	109.1	C34—C43—H43	106.5
C11—C10—H10B	109.1	C42—C44—H44A	109.5
C3—C10—H10B	109.1	C42—C44—H44B	109.5
H10A—C10—H10B	107.9	H44A—C44—H44B	109.5
C12—C11—C10	123.0 (7)	C42—C44—H44C	109.5
C12—C11—H11	118.5	H44A—C44—H44C	109.5
C10—C11—H11	118.5	H44B—C44—H44C	109.5
C11—C12—C13	122.7 (7)	C46—C45—C43	114.8 (6)
C11—C12—C14	117.8 (7)	C46—C45—H45A	108.6
C13—C12—C14	119.5 (7)	C43—C45—H45A	108.6
C12—C13—C15	113.2 (6)	C46—C45—H45B	108.6
C12—C13—C4	112.6 (6)	C43—C45—H45B	108.6
C15—C13—C4	111.5 (6)	H45A—C45—H45B	107.5
C12—C13—H13	106.3	C47—C46—C45	113.6 (6)
C15—C13—H13	106.3	C47—C46—H46A	108.8
C4—C13—H13	106.3	C45—C46—H46A	108.8
C12—C14—H14A	109.5	C47—C46—H46B	108.8
C12—C14—H14B	109.5	C45—C46—H46B	108.8
H14A—C14—H14B	109.5	H46A—C46—H46B	107.7
C12—C14—H14C	109.5	C48'—C47—C46	118.7 (9)
H14A—C14—H14C	109.5	C46—C47—C48	110.2 (10)
H14B—C14—H14C	109.5	C48'—C47—C56	106.5 (13)
C16—C15—C13	113.7 (6)	C46—C47—C56	112.9 (6)
C16—C15—H15A	108.8	C48—C47—C56	113.8 (11)
C13—C15—H15A	108.8	C48'—C47—H47	105.0
C16—C15—H15B	108.8	C46—C47—H47	106.4
C13—C15—H15B	108.8	C48—C47—H47	106.4
H15A—C15—H15B	107.7	C56—C47—H47	106.4
C17—C16—C15	113.2 (6)	C48'—C47—H47'	105.9
C17—C16—H16A	108.9	C46—C47—H47'	105.9
C15—C16—H16A	108.9	C48—C47—H47'	107.4
C17—C16—H16B	108.9	C56—C47—H47'	105.9
C15—C16—H16B	108.9	C57—C48—C49	115 (2)
H16A—C16—H16B	107.8	C57—C48—C47	129 (3)
C18—C17—C16	114.8 (6)	C49—C48—C47	110.1 (15)
C18—C17—C26	109.8 (5)	C50—C49—C48	128 (2)
C16—C17—C26	114.0 (6)	C50—C49—H49A	105.4
C18—C17—H17	105.8	C48—C49—H49A	105.4
C16—C17—H17	105.8	C50—C49—H49B	105.4
C26—C17—H17	105.8	C48—C49—H49B	105.4
C27—C18—C19	122.2 (7)	H49A—C49—H49B	106.0
C27—C18—C17	123.3 (7)	C49'—C48'—C47	125 (2)
C19—C18—C17	114.5 (7)	C49'—C48'—C57'	122 (3)
C18—C19—C20	111.7 (7)	C47—C48'—C57'	113.0 (17)
C18—C19—H19A	109.3	C48'—C49'—C50	109.4 (19)
C20—C19—H19A	109.3	C48'—C49'—H49'	125.3
C18—C19—H19B	109.3	C50—C49'—H49'	125.3
C20—C19—H19B	109.3	C49—C50—C51	117.3 (12)
H19A—C19—H19B	107.9	C51—C50—C49'	109.2 (11)
C19—C20—C21	109.9 (6)	C49—C50—H50A	108.0
C19—C20—H20A	109.7	C51—C50—H50A	108.0
C21—C20—H20A	109.7	C49'—C50—H50A	98.8
C19—C20—H20B	109.7	C49—C50—H50B	108.0
C21—C20—H20B	109.7	C51—C50—H50B	108.0
H20A—C20—H20B	108.2	C49'—C50—H50B	124.4
C20—C21—C22	113.2 (6)	H50A—C50—H50B	107.2
C20—C21—C26	110.8 (6)	C49—C50—H50C	93.3
C22—C21—C26	117.2 (5)	C51—C50—H50C	109.8
C20—C21—H21	104.8	C49'—C50—H50C	109.8
C22—C21—H21	104.8	H50A—C50—H50C	120.4
C26—C21—H21	104.8	C49—C50—H50D	116.6
C29—C22—C23	109.7 (7)	C51—C50—H50D	109.8
C29—C22—C28	108.2 (6)	C49'—C50—H50D	109.8
C23—C22—C28	106.1 (6)	H50C—C50—H50D	108.3
C29—C22—C21	110.0 (6)	C50—C51—C52	114.6 (6)
C23—C22—C21	107.4 (6)	C50—C51—C56	110.1 (6)
C28—C22—C21	115.2 (6)	C52—C51—C56	118.8 (6)
O2—C23—C24	120.5 (8)	C50—C51—H51	103.8
O2—C23—C22	122.6 (7)	C52—C51—H51	103.8
C24—C23—C22	116.9 (6)	C56—C51—H51	103.8
C23—C24—C25	111.0 (6)	C53—C52—C51	108.9 (5)
C23—C24—H24A	109.4	C53—C52—C58	107.5 (6)
C25—C24—H24A	109.4	C51—C52—C58	111.9 (5)
C23—C24—H24B	109.4	C53—C52—C59	109.6 (6)
C25—C24—H24B	109.4	C51—C52—C59	109.6 (7)
H24A—C24—H24B	108.0	C58—C52—C59	109.3 (6)
C24—C25—C26	113.8 (6)	O4—C53—C54	122.9 (7)
C24—C25—H25A	108.8	O4—C53—C52	121.4 (7)
C26—C25—H25A	108.8	C54—C53—C52	115.6 (6)
C24—C25—H25B	108.8	C53—C54—C55	112.5 (7)
C26—C25—H25B	108.8	C53—C54—H54A	109.1
H25A—C25—H25B	107.7	C55—C54—H54A	109.1
C30—C26—C25	110.2 (5)	C53—C54—H54B	109.1
C30—C26—C21	114.7 (5)	C55—C54—H54B	109.1
C25—C26—C21	108.2 (6)	H54A—C54—H54B	107.8
C30—C26—C17	109.1 (6)	C54—C55—C56	112.4 (6)
C25—C26—C17	108.1 (5)	C54—C55—H55A	109.1
C21—C26—C17	106.3 (5)	C56—C55—H55A	109.1
C18—C27—H27A	120.0	C54—C55—H55B	109.1
C18—C27—H27B	120.0	C56—C55—H55B	109.1
H27A—C27—H27B	120.0	H55A—C55—H55B	107.9
C22—C28—H28A	109.5	C60—C56—C55	111.3 (6)
C22—C28—H28B	109.5	C60—C56—C47	109.6 (6)
H28A—C28—H28B	109.5	C55—C56—C47	108.0 (5)
C22—C28—H28C	109.5	C60—C56—C51	113.8 (6)
H28A—C28—H28C	109.5	C55—C56—C51	107.9 (6)
H28B—C28—H28C	109.5	C47—C56—C51	106.1 (5)
C22—C29—H29A	109.5	C48—C57—H57A	120.0
C22—C29—H29B	109.5	C48—C57—H57B	120.0
H29A—C29—H29B	109.5	H57A—C57—H57B	120.0
C22—C29—H29C	109.5	C48'—C57'—H57C	109.5
H29A—C29—H29C	109.5	C48'—C57'—H57D	109.5
H29B—C29—H29C	109.5	H57C—C57'—H57D	109.5
C26—C30—H30A	109.5	C48'—C57'—H57E	109.5
C26—C30—H30B	109.5	H57C—C57'—H57E	109.5
H30A—C30—H30B	109.5	H57D—C57'—H57E	109.5
C26—C30—H30C	109.5	C52—C58—H58A	109.5
H30A—C30—H30C	109.5	C52—C58—H58B	109.5
H30B—C30—H30C	109.5	H58A—C58—H58B	109.5
O3—C31—C36	120.0 (7)	C52—C58—H58C	109.5
O3—C31—C32	121.2 (7)	H58A—C58—H58C	109.5
C36—C31—C32	118.7 (6)	H58B—C58—H58C	109.5
C37—C32—C31	108.7 (7)	C52—C59—H59A	109.5
C37—C32—C38	108.3 (6)	C52—C59—H59B	109.5
C31—C32—C38	105.8 (6)	H59A—C59—H59B	109.5
C37—C32—C33	108.2 (6)	C52—C59—H59C	109.5
C31—C32—C33	111.9 (6)	H59A—C59—H59C	109.5
C38—C32—C33	113.8 (6)	H59B—C59—H59C	109.5
C40—C33—C34	109.9 (5)	C56—C60—H60A	109.5
C40—C33—C32	112.0 (6)	C56—C60—H60B	109.5
C34—C33—C32	119.3 (6)	H60A—C60—H60B	109.5
C40—C33—H33	104.8	C56—C60—H60C	109.5
C34—C33—H33	104.8	H60A—C60—H60C	109.5
C32—C33—H33	104.8	H60B—C60—H60C	109.5
			
O1—C1—C2—C8	−30.2 (10)	C37—C32—C33—C34	−151.3 (6)
C6—C1—C2—C8	151.8 (7)	C31—C32—C33—C34	−31.6 (8)
O1—C1—C2—C7	85.7 (8)	C38—C32—C33—C34	88.2 (8)
C6—C1—C2—C7	−92.3 (8)	C40—C33—C34—C35	176.9 (6)
O1—C1—C2—C3	−151.7 (7)	C32—C33—C34—C35	45.7 (8)
C6—C1—C2—C3	30.3 (9)	C40—C33—C34—C39	55.5 (8)
C1—C2—C3—C10	−162.0 (7)	C32—C33—C34—C39	−75.7 (8)
C8—C2—C3—C10	75.2 (8)	C40—C33—C34—C43	−65.3 (7)
C7—C2—C3—C10	−44.7 (10)	C32—C33—C34—C43	163.5 (6)
C1—C2—C3—C4	−30.3 (9)	C33—C34—C35—C36	−59.7 (8)
C8—C2—C3—C4	−153.0 (7)	C39—C34—C35—C36	63.3 (8)
C7—C2—C3—C4	87.1 (8)	C43—C34—C35—C36	−176.6 (6)
C10—C3—C4—C5	178.0 (6)	O3—C31—C36—C35	136.2 (7)
C2—C3—C4—C5	45.0 (8)	C32—C31—C36—C35	−47.7 (9)
C10—C3—C4—C9	54.7 (8)	C34—C35—C36—C31	61.9 (8)
C2—C3—C4—C9	−78.2 (8)	C34—C33—C40—C41	47.9 (8)
C10—C3—C4—C13	−65.2 (8)	C32—C33—C40—C41	−177.1 (6)
C2—C3—C4—C13	161.9 (6)	C33—C40—C41—C42	−13.5 (10)
C9—C4—C5—C6	64.8 (8)	C40—C41—C42—C43	−3.6 (11)
C3—C4—C5—C6	−59.2 (8)	C40—C41—C42—C44	174.9 (7)
C13—C4—C5—C6	−174.6 (6)	C41—C42—C43—C45	−141.8 (7)
O1—C1—C6—C5	135.6 (7)	C44—C42—C43—C45	39.6 (8)
C2—C1—C6—C5	−46.4 (9)	C41—C42—C43—C34	−14.6 (9)
C4—C5—C6—C1	61.1 (9)	C44—C42—C43—C34	166.9 (6)
C4—C3—C10—C11	47.6 (9)	C35—C34—C43—C42	164.7 (6)
C2—C3—C10—C11	−176.5 (7)	C33—C34—C43—C42	47.6 (7)
C3—C10—C11—C12	−12.6 (13)	C39—C34—C43—C42	−75.0 (7)
C10—C11—C12—C13	−3.6 (14)	C35—C34—C43—C45	−67.2 (7)
C10—C11—C12—C14	175.7 (9)	C33—C34—C43—C45	175.7 (5)
C11—C12—C13—C15	−143.3 (8)	C39—C34—C43—C45	53.0 (7)
C14—C12—C13—C15	37.5 (11)	C42—C43—C45—C46	−88.7 (7)
C11—C12—C13—C4	−15.7 (11)	C34—C43—C45—C46	143.6 (6)
C14—C12—C13—C4	165.0 (8)	C43—C45—C46—C47	−157.6 (6)
C5—C4—C13—C12	164.8 (6)	C45—C46—C47—C48'	−78.0 (19)
C9—C4—C13—C12	−73.5 (8)	C45—C46—C47—C48	−75.0 (17)
C3—C4—C13—C12	48.4 (8)	C45—C46—C47—C56	156.4 (6)
C5—C4—C13—C15	−66.8 (8)	C48'—C47—C48—C57	−152 (21)
C9—C4—C13—C15	55.0 (8)	C46—C47—C48—C57	45 (4)
C3—C4—C13—C15	176.9 (6)	C56—C47—C48—C57	173 (3)
C12—C13—C15—C16	−90.1 (8)	C48'—C47—C48—C49	0(16)
C4—C13—C15—C16	141.7 (7)	C46—C47—C48—C49	−163.3 (18)
C13—C15—C16—C17	−158.1 (6)	C56—C47—C48—C49	−35 (3)
C15—C16—C17—C18	−73.5 (8)	C57—C48—C49—C50	169 (2)
C15—C16—C17—C26	158.6 (6)	C47—C48—C49—C50	13 (4)
C16—C17—C18—C27	−7.1 (10)	C46—C47—C48'—C49'	173 (2)
C26—C17—C18—C27	122.9 (8)	C56—C47—C48'—C49'	−58 (3)
C16—C17—C18—C19	173.6 (6)	C46—C47—C48'—C57'	−8(3)
C26—C17—C18—C19	−56.4 (8)	C56—C47—C48'—C57'	121 (2)
C27—C18—C19—C20	−125.1 (9)	C47—C48'—C49'—C50	53 (4)
C17—C18—C19—C20	54.2 (9)	C57'—C48'—C49'—C50	−126 (3)
C18—C19—C20—C21	−54.6 (9)	C48—C49—C50—C51	−17 (3)
C19—C20—C21—C22	−166.2 (6)	C48—C49—C50—C49'	−81 (6)
C19—C20—C21—C26	59.9 (8)	C48'—C49'—C50—C49	74 (7)
C20—C21—C22—C29	60.8 (8)	C48'—C49'—C50—C51	−48 (3)
C26—C21—C22—C29	−168.4 (6)	C49—C50—C51—C52	178.4 (13)
C20—C21—C22—C23	−179.8 (6)	C49'—C50—C51—C52	−165.9 (11)
C26—C21—C22—C23	−49.0 (8)	C49—C50—C51—C56	41.4 (15)
C20—C21—C22—C28	−61.8 (8)	C49'—C50—C51—C56	57.1 (12)
C26—C21—C22—C28	69.0 (8)	C50—C51—C52—C53	−177.4 (7)
C29—C22—C23—O2	−12.1 (10)	C56—C51—C52—C53	−44.3 (8)
C28—C22—C23—O2	104.6 (9)	C50—C51—C52—C58	−58.7 (9)
C21—C22—C23—O2	−131.6 (8)	C56—C51—C52—C58	74.4 (8)
C29—C22—C23—C24	169.7 (7)	C50—C51—C52—C59	62.8 (9)
C28—C22—C23—C24	−73.6 (8)	C56—C51—C52—C59	−164.1 (7)
C21—C22—C23—C24	50.2 (8)	C51—C52—C53—O4	−136.3 (9)
O2—C23—C24—C25	127.2 (8)	C58—C52—C53—O4	102.3 (9)
C22—C23—C24—C25	−54.5 (9)	C59—C52—C53—O4	−16.4 (11)
C23—C24—C25—C26	54.9 (9)	C51—C52—C53—C54	47.0 (9)
C24—C25—C26—C30	73.9 (8)	C58—C52—C53—C54	−74.4 (8)
C24—C25—C26—C21	−52.2 (7)	C59—C52—C53—C54	166.9 (8)
C24—C25—C26—C17	−166.9 (6)	O4—C53—C54—C55	127.5 (9)
C20—C21—C26—C30	59.7 (7)	C52—C53—C54—C55	−55.8 (9)
C22—C21—C26—C30	−72.2 (8)	C53—C54—C55—C56	58.3 (9)
C20—C21—C26—C25	−176.8 (6)	C54—C55—C56—C60	73.9 (8)
C22—C21—C26—C25	51.3 (7)	C54—C55—C56—C47	−165.8 (6)
C20—C21—C26—C17	−60.9 (7)	C54—C55—C56—C51	−51.6 (8)
C22—C21—C26—C17	167.2 (6)	C48'—C47—C56—C60	−68.1 (13)
C18—C17—C26—C30	−66.9 (7)	C46—C47—C56—C60	63.9 (8)
C16—C17—C26—C30	63.5 (7)	C48—C47—C56—C60	−62.8 (15)
C18—C17—C26—C25	173.2 (6)	C48'—C47—C56—C55	170.6 (12)
C16—C17—C26—C25	−56.3 (7)	C46—C47—C56—C55	−57.5 (8)
C18—C17—C26—C21	57.3 (7)	C48—C47—C56—C55	175.8 (14)
C16—C17—C26—C21	−172.3 (6)	C48'—C47—C56—C51	55.1 (13)
O3—C31—C32—C37	−32.7 (9)	C46—C47—C56—C51	−172.9 (6)
C36—C31—C32—C37	151.3 (7)	C48—C47—C56—C51	60.4 (15)
O3—C31—C32—C38	83.4 (8)	C50—C51—C56—C60	58.7 (8)
C36—C31—C32—C38	−92.6 (8)	C52—C51—C56—C60	−76.3 (8)
O3—C31—C32—C33	−152.1 (7)	C50—C51—C56—C55	−177.3 (6)
C36—C31—C32—C33	31.9 (9)	C52—C51—C56—C55	47.7 (8)
C37—C32—C33—C40	78.4 (8)	C50—C51—C56—C47	−61.9 (7)
C31—C32—C33—C40	−161.9 (6)	C52—C51—C56—C47	163.2 (6)
C38—C32—C33—C40	−42.1 (8)		
